# Small Molecular-Sized Artesunate Attenuates Ocular Neovascularization via VEGFR2, PKCα, and PDGFR Targets

**DOI:** 10.1038/srep30843

**Published:** 2016-08-02

**Authors:** Yao Zong, Yongguang Yuan, Xiaobing Qian, Zhen Huang, Wei Yang, Leilei Lin, Qishan Zheng, Yujie Li, Huining He, Qianying Gao

**Affiliations:** 1 State Key Laboratory of Ophthalmology, Zhongshan Ophthalmic Center, Sun Yat-sen University, Guangzhou 510060, China

## Abstract

Ocular neovascularization (NV) is the primary cause of blindness in many ocular diseases. Large molecular weight anti- vascular endothelial growth factor (VEGF) protein drugs, such as Avastin and Lucentis, have saved the vision of millions. However, approximately 20–30% of patients respond poorly to anti-VEGF treatment. We found that artesunate (ART), a small molecular derivative of artemisinin, had a significant inhibitory effect on ocular NV by downregulating the expression of VEGFR2, PKCα, and PDGFR. ART significantly inhibited retinal NV in rabbits and macular edema in monkeys with greater anterior chamber penetrability and more durable efficacy than Avastin. Our pilot study showed that intravitreal injection of 80 μg ART significantly inhibited iris and corneal NV in a severe retinal detachment case. Our results suggest that ART might be a potential persistent small-molecule drug to manage ocular NV via multi-targets.

Ocular neovascularization (NV) diseases, such as diabetic retinopathy (DR) and age-related macular degeneration (AMD), constitute the leading cause of severe vision loss and irreversible blindness in developing and developed countries^1^. Substantial evidence indicates that vascular endothelial growth factor (VEGF) plays an important role in the pathogenesis of ocular NV^2^,^3^. Large molecular weight anti-VEGF protein drugs, such as Avastin (bevacizumab, 149kDa) and Lucentis (ranibizumab, 48kDa), represent the most widely used therapies that appear to have the potential to enable many ocular NV patients to obtain a sustained gain of vision^4^,^5^. However, resistance to these drugs remains a problem; approximately 20–30% of patients respond poorly to anti-VEGF treatment^6^.

Artemisinin is extracted from the Chinese herb qinghao, and its discovery by Chinese chemists led to a Lasker Award in 2011^7^ and a Noble prize in 2015. It is a remarkable anti-malarial^8^ and anti-tumor drug^9^. The derivatives of artemisinin, such as artesunate (ART) (384Da)^10^,^11^, dihydroartemisinin (DI)^12^, arteeter, artemether and artemisone^9^, appear to be more potent than the substance itself^11^. The known mechanisms of artemisinin and its derivatives in inhibiting angiogenesis include downregulating several growth factors (VEGF, fibroblast growth factor or FGF, hypoxia-inducible factor 1-alpha or HIF-1α, and angiopoietin 1 or Ang-1)^13^,^14^,^15^,^16^, upregulating angiogenesis inhibitors^17^,^18^, depleting the levels of the flt-1 and KDR/flk-1-receptors^19^,^20^, and inducing apoptosis of vascular endothelial cells^19^,^21^. In the science of ophthalmology, ART has been reported to inhibit corneal NV in animal models by inducing reactive oxygen species (ROS)-dependent apoptosis^22^. However, it has never been deployed for use in retinal NV.

In this study, we provided animal evidence that ART might be a potential drug candidate to manage ocular NV, especially retinal NV, and thus revealed a novel mechanism of action. ART significantly inhibited iris and retinal NV in rabbits and iris NV and macular edema in monkeys. It had greater anterior chamber penetrability and more durable efficacy than Avastin without resulting in significant ocular structural disorders during the follow-up period.

## Results

### ART attenuates retinal neovascularization in rabbit models

**Figure 1:**
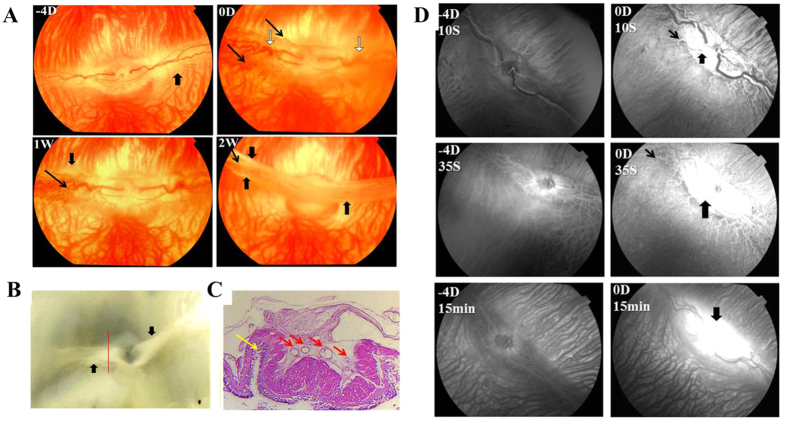
Fundus and FFA of the model group at different time points. At day 0, vascular dilatation and tortuosity (white arrows in **A**), NV at the margin of medullary wings (thin black arrows in **A**), and fluorescein leakage (thick black arrows in **D**) from the disc and medullary were found in rabbit eyes injected with VEGF165+bFGF. Tractional folds of the medullary fibers (thick black arrows in **A** and **B**) appeared at 1 week, and tractional retinal detachments occurred at 2 weeks (yellow arrows in **C**), along with vascular crowding (red arrows in **C**).

In our rabbit models, intravitreal injection of VEGF_165_+ basic FGF(bFGF) (2 μg VEGF_165_, 2 μg bFGF) resulted in vascular dilatation and tortuosity, and fluorescein leakage at the optic disc and medullary wings (Fig. 1) at day 0 in all groups.

We then intravitreally injected 10, 20, and 40 μg/mL ART into six model eyes (two rabbits in each dosage group) and found that 20 and 40 μg/mL ART significantly inhibited retinal NV (Fig. S1). We therefore selected 20 μg/mL ART for further study. Interestingly, this is also the anti-malarial serum drug concentration.

**Figure 2:**
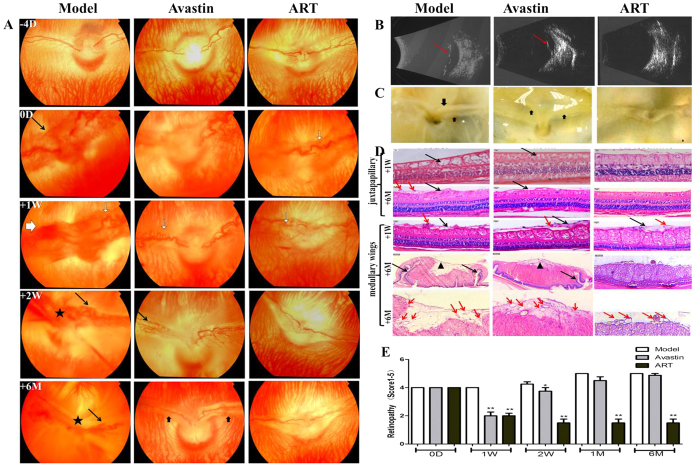
The effect of ART on rabbit retinal vascular disorders induced by VEGF165+bFGF. (**A–C**) ART and Avastin significantly diminished vascular dilatation and tortuosity (white arrows in A) and NV (black arrows in **A**) at 1 week. However, NV and fluorescein leakage recurred in the Avastin group at 2 weeks and finally resulted in tractional detachment of the retina (thick black arrows in **A** and **C**, red arrows in **B**). The tractional retinal detachments occurred at 2 weeks in the model group (★ in **A**). No visible neovascular membrane occurred in the ART group for 6 months. (**D**) H&E staining of cross-sections of the medullary wings (medullary) and superior to the optic disc (juxtapapillary). ART and Avastin significantly inhibited retinal edema (black arrows) at 1 week compared to the model group but did not noticeably reduce epiretinal fibrovascular membranes (red arrows). After 6 months, epiretinal fibrosis (▲) and retinal folds (black arrows) appeared in the model and Avastin groups, along with vascular crowding, which was not found in the ART group. The inner retina was disorganized (black arrows) in the model group; this did not occur in the ART or Avastin groups. (**E**) Grade score for retinal vascular disorders. All fundus and FFA photographs of rabbits were evaluated independently and in a masked manner by two observers and were scored using the five grades of retinal NV. The grade scores were used for ANOVA analysis, and differences with a value of p < 0.05 were considered significant.

**Figure 3:**
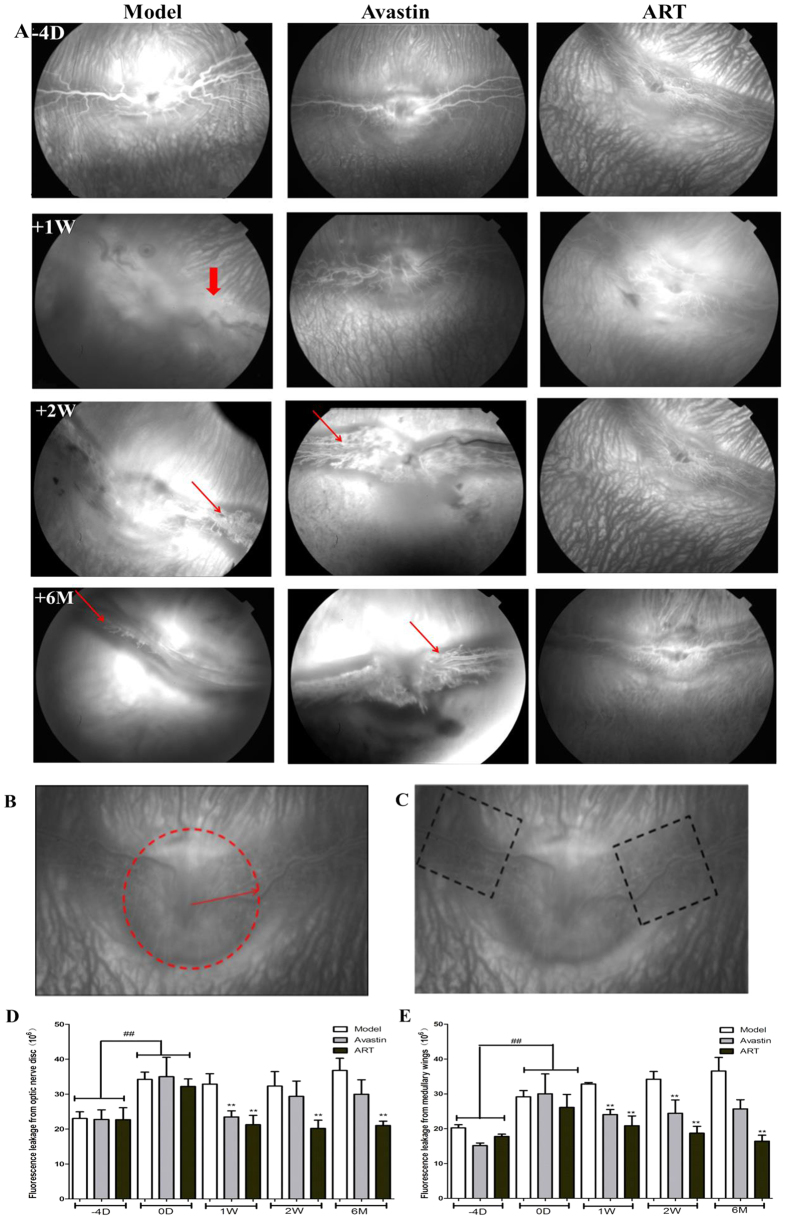
The effect of ART on rabbit retinal vascular leakage induced by VEGF165+bFGF. (**A**) ART and Avastin significantly reduced NV (thin red arrows) and fluorescein leakage (thick red arrows) at 1 week. However, NV and fluorescein leakage reccurred in the Avastin group at 2 weeks. No visible neovascular membrane occurred in the ART group for 6 months. (**B**,**C**) Diagram for analysis on the fluorescein leakage intensity from optic disc and both medullary wings in the late phase of FFA. The fluorescein leakage intensity in the selected areas was measured by Image J, and the mean fluorescein leakage intensity was used for statistical analysis. (**D**,**E**) Fluorescein leakage intensity from optic disc and medullary wings. Significant differences were determined using an ANOVA and SPSS 20.0 software (* and **denote p < 0.05 and p < 0.01 compared to the model group, and ^#^and ^##^denote p < 0.05 and p < 0.01 compared to the baseline).

After 1 week, the leakage persisted in the model group, and a well-developed neovascular membrane was visible on color fundus photography (Fig. 2A) and fundus fluorescein angiography (FFA) (Fig. 3A). All the rabbit eyes in the model group ended in tractional detachment of the retina at 2 weeks, with the pale optic disc and medullary wings being irregularly elevated (Figs 1B,C and 2B–D).

In groups treated with ART or Avastin, vascular dilatation, tortuosity, and fluorescein leakage decreased significantly during the first 2 weeks after a single injection of ART or Avastin (Figs 2A and 3A). However, the effect of Avastin weakened after 2 weeks, and the rabbit eyes ended in tractional detachment of the retina. In contrast, VEGF_165_+bFGF-induced retinal vascular disorders were completely blocked by ART for 6 months (Fig. 2). In groups with ART and Avastin, fluorescein leakage decreased significantly at 2 weeks after injection; however, in the Avastin group, the intensity of fluorescence leakage increased again after 2 weeks (Fig. 3).

Hematoxylin and eosin (H&E) staining further showed that ART inhibits retinal edema and epiretinal fibrovascular membranes (Fig. 2D). After 1 week, edema appeared in the retina superior to the optic disc in the model group, while retinal edema was obviously alleviated in the ART and Avastin groups. After 6 months, retinal edema had faded, leaving inner retinal structural disorders in the model group. Epiretinal fibrovascular membranes appeared in the model and Avastin groups. The retinal folds at the upper and lower edges of the medullary wings appeared in the model and Avastin groups, along with vascular crowding, which was not found in the ART group. In summary, ART had more durable efficacy than Avastin in inhibiting retinal NV.

### ART reduced the expression of VEGFR2, PKCα, and PDGFR in rabbit models

**Figure 4:**
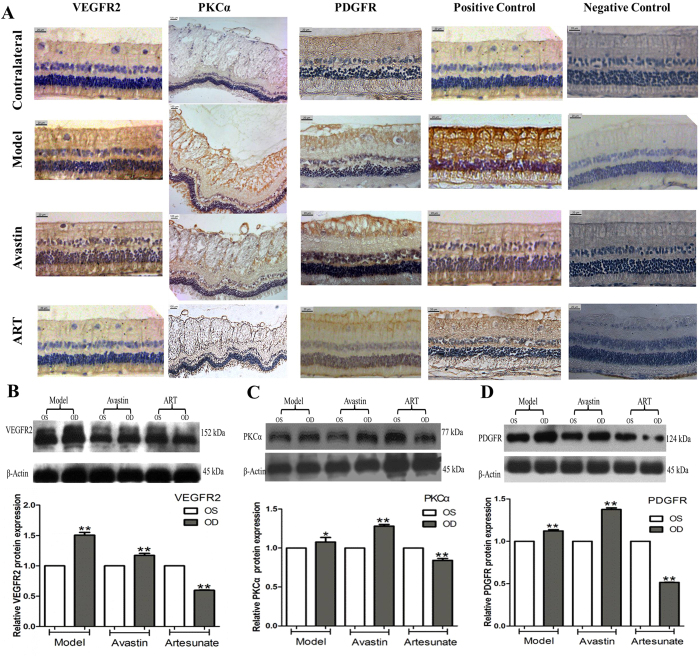
ART inhibited the expression of VEGFR2, PKCα, and PDGFR in rabbit retina. (**A**) IHC of rabbit retina tissue. The retina tissue in the treated eyes of groups Model and Avastin showed stronger expression of VEGFR2, PKCα and PDGFR than in the contralateral eyes; however, the expression was significantly lower in the treated eyes of group ART. (**B**–**D**) Western Blotting of rabbit retina tissue. The names of proteins are shown on the left, and the molecular weight sizes of proteins are shown on the right, respectively. The expression of VEGFR2, PKCα, and PDGFR in the treated eyes of the Model and Avastin groups was stronger compared that in the contralateral eyes; however, the expression was significantly lower in the ART group. Three rabbits from each group were harvested for IHC analysis, and another three rabbits from each group for Western blotting at 1 month. Each test was repeated three times and the mean intensity was used in the analysis. The blots were representative of three independently performed experiments. (One-way ANOVA, * and **denote P  <  0.05 and P  <  0.01 compared with left eye of the same rabbit)

We further confirmed that ART suppressed protein expression of VEGF receptor 2 (VEGFR2), protein kinase Cα isoenzyme (PKCα), and platelet-derived growth factor receptor (PDGFR) in the retinal tissue of rabbits treated for 1 month through immunohistochemistry (IHC) (Fig. 4A) and western blotting (Fig. 4B–D, Fig. S2). The expression of VEGFR2, PKCα, and PDGFR in the treated eyes of the model and Avastin groups was stronger compared to that in contralateral eyes; however, the expression was significantly lower in the ART group. The results suggest that ART suppresses the expression of VEGFR2, PKCα, and PDGFR in rabbit retinas.

### ART inhibits iris neovascularization and fluorescein leakage into the anterior chamber in rabbits

**Figure 5:**
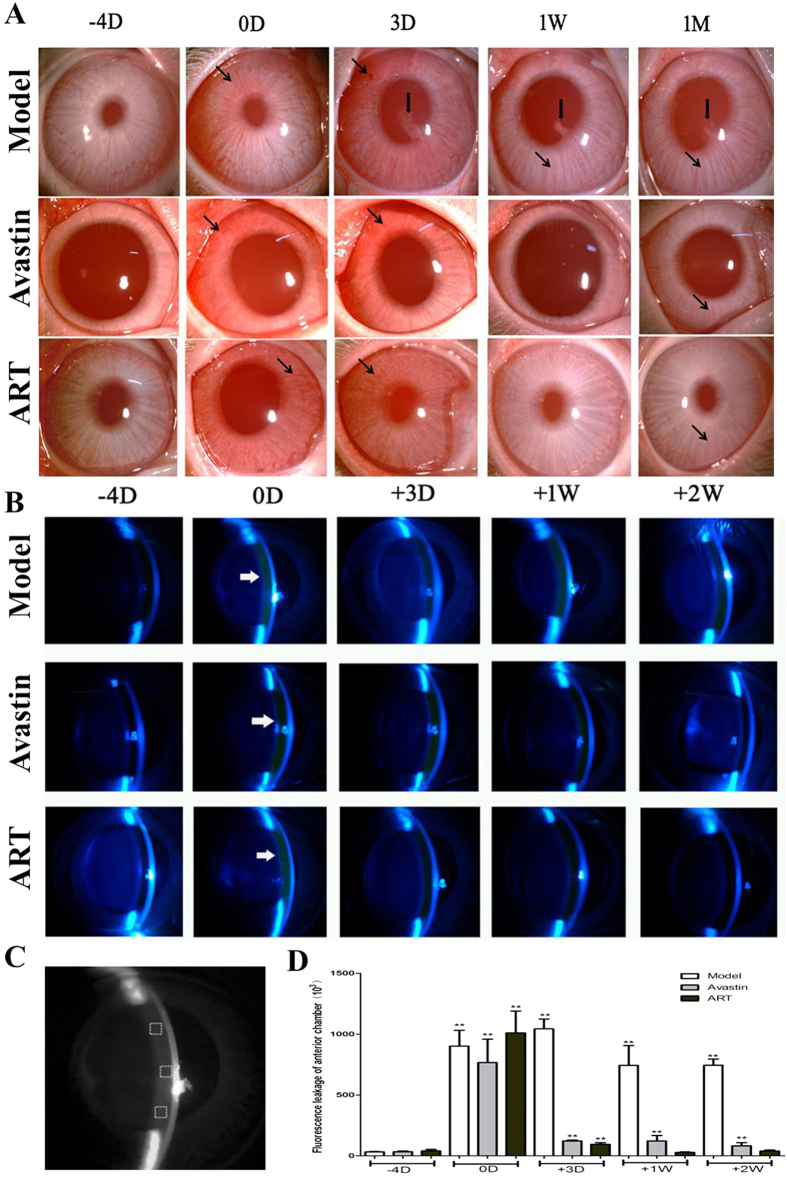
The effect of ART on iris NV and fluorescein leakage in the anterior chamber. (**A**) Iris NV (black arrows) lasted for 1 month in the model group, with the presence of posterior synechia (thick black arrows). Both gradually vanished within 1 week in the ART and Avastin groups. (**B–D**) The intensity of fluorescein leakage in the anterior chamber (white arrows in B) was significantly stronger compared to baseline levels in the model group, and returned to baseline levels within 1week in the ART group and within 2 weeks in the Avastin group (**D)**. (**C**) Principle diagram for quantitative evaluation of the intensity of fluorescence leakage in the anterior chamber. * and **denote p < 0.05 and p < 0.01 compared with baseline levels.

Because it is a small molecule, ART can inhibit iris NV and fluorescein leakage into the anterior chamber due to greater penetrability. Iris NV lasted for 1 month in the model group and vanished gradually within 1 week in the ART and Avastin groups, without posterior synechia (Fig. 5A).

The intensity of fluorescein leakage in the anterior chamber was significantly higher than the baseline level in the model group but returned to the baseline level within 1 week in the ART group and within 2 weeks in the Avastin group (Fig. 5B–D).

### ART inhibits fluorescein leakage and macular edema in monkeys

**Figure 6:**
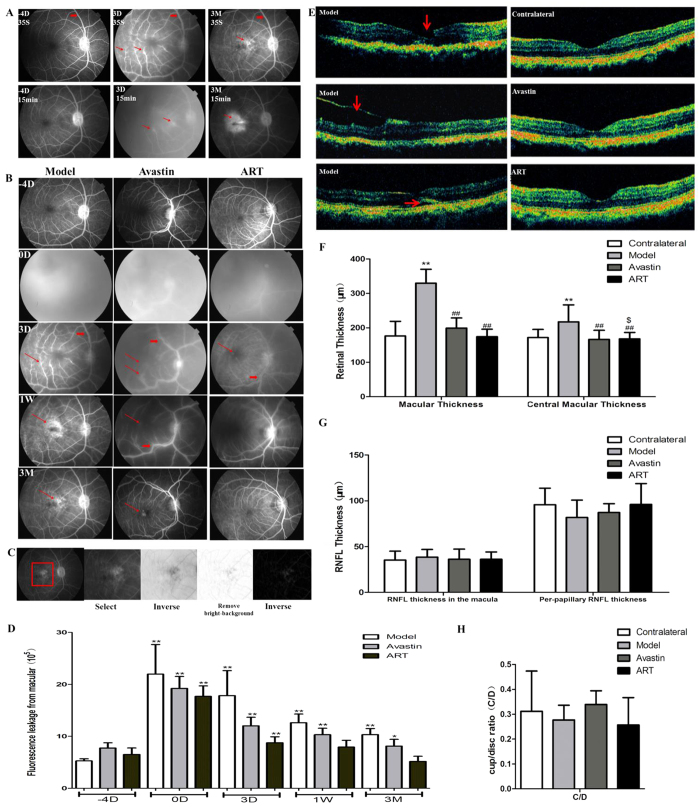
ART inhibited fluorescein leakage and molecular edema in monkey models. (**A**) FFA images of the model group. Retinal vascular dilatation and tortuosity (thick red arrows) and fluorescein leakage (thin red arrows) from the macula occurred at day 0 in the model group. (**B**) FFA images of all groups. The fluorescein leakage in the macular area lasted for 3 months in the model and Avastin groups (thin red arrows) and decreased gradually in the ART group. (**C**) Principle diagram for quantitative evaluation of the intensity of fluorescence leakage of the macula. Three FFA images of each monkey were taken at 15 min, a square-shaped area of the macula in the same size was selected, and the fluorescence intensity in this area was measured after background elimination. (**D**) Fluorescein leakage intensity of the macula. * and **denote p < 0.05 and p < 0.01 compared to the baseline level. (**E**) OCT. The macular retinal tear (red arrows), epiretinal membranes (red arrows), and retinal detachment (red arrows) appeared in the model group and were not found in the Avastin and ART groups. (**F**) Retinal thickness. The mean retinal thickness of the macular significantly increased in the model group compared to the contralateral eye and decreased in the ART and Avastin groups. (**G,H**) RNFL and C/D. Significant differences were determined using a *t* test or an ANOVA with SPSS 20.0 software. * and **denote p < 0.05 and p < 0.01, compared to the control group; ^#^and ^##^denote p < 0.05 and p < 0.01compared to the model group; ^$^ and ^$$^denote p < 0.05 and p < 0.01 compared to the Avastin group.

A similar protocol was conducted in monkey models. The fluorescein leakage of the macula occurred at day 0 and lasted for 3 months in the model and Avastin groups and returned to the baseline level within 1 week in the ART group (Fig. 6A–D). Optical coherence tomography (OCT) showed a macular retinal tear, an epiretinal membrane, and retinal detachment in the model group, which were not found in the Avastin and ART groups (Fig. 6E). The mean retinal thickness of the macula increased significantly in the model group and showed no significant difference in the Avastin and ART groups compared to the contralateral eyes (Fig. 6F). No significant difference in retinal nerve fiber layer (RNFL) thickness or cup/disc ratio (C/D) was found in the Avastin and ART groups from the contralateral eyes (Fig. 6G,H).

### ART inhibits iris neovascularization and fluorescein leakage into the anterior chamber in monkeys

**Figure 7:**
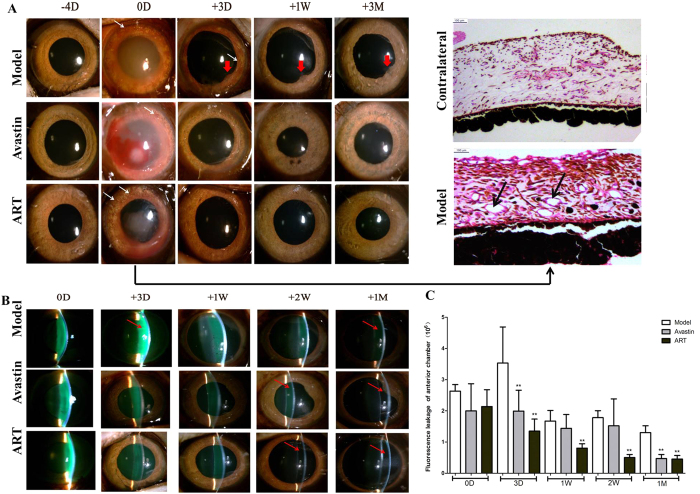
Effect of ART on iris NV and fluorescein leakage into the anterior chamber in monkey models. (**A**) Iris NV appeared at day 0, as revealed by a photograph of the iris (white arrows) and H&E staining (black arrows), lasted for 3 days in the model group, with the presence of posterior synechia (thick red arrows). It vanished completely in the ART and Avastin groups by day 3. (**B,C**) Fluorescein leakage (red arrows) in the anterior chamber lasted for 1 month in the model group and was essentially eliminated within 2 weeks in the ART group and within 1 month in the Avastin group. * and **denote p < 0.05 and p < 0.01 compared to the model group.

Iris NV of monkeys lasted for 3 days in the model group but vanished in the ART and Avastin groups in the first 3 days, without posterior synechia (Fig. 7A). The fluorescein leakage into the anterior chamber lasted for 1 month in the model group and was essentially eliminated in 2 weeks in the ART group and in 1 month in the Avastin group (Fig. 7B,C).

### ART inhibited corneal neovascularization in a case of severe retinal detachment

**Figure 8:**
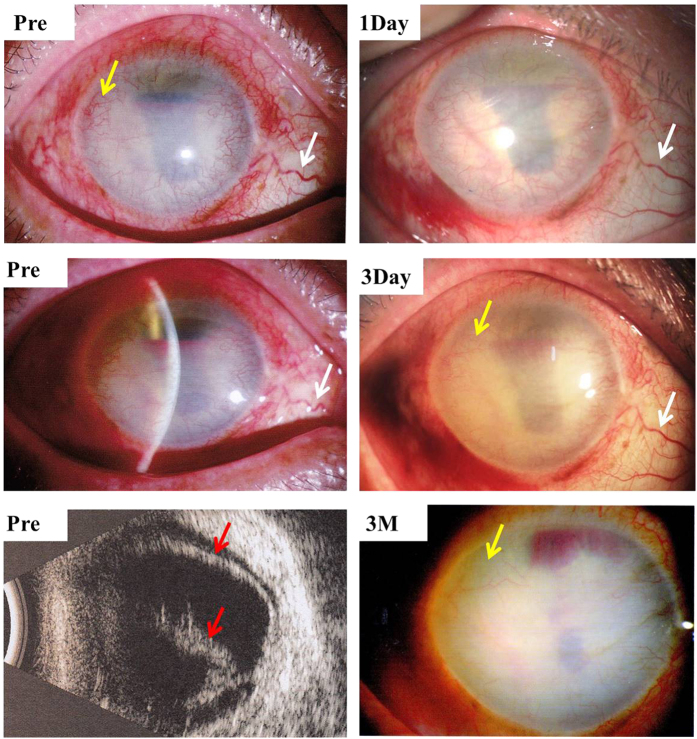
Intravitreal injection of 80μg ART inhibited corneal NV in a case of severe retinal detachment (red arrows) and corneal NV (yellow arrows). Dilatation and tortuosity of conjunctival vessels were noticeably alleviated (white arrows) 1 day after treatment, and corneal NV (yellow arrows) decreased after 3 days and vanished mostly after 3 months.

Our pilot study showed that intravitreal injection of 80 μg ART significantly inhibited iris and corneal NV in a severe retinal detachment case with no light perception 3 months after treatment (Fig. 8).

### Safety evaluation of intravitreal injection of ART

In VEGF_165_+bFGF-induced retinal NV rabbit models, H&E staining revealed no significant ocular structural disorders, including of the cornea, iris, and ciliary body 6 months after treatment with 20 μg ART compared to contralateral eyes (Fig. S3). In VEGF_165_+bFGF-induced retinal NV monkey models, corneal endothelium, electroretinogram (ERG), vessel diameter, and oxygen saturation of the retina at 3 and 6 months did not show significant changes between the treated and contralateral eyes after a 20 μg ART injection (Fig. S3). ART has a selective killing effect on proliferative cells with a low drug dosage for anti-angiogenesis (about 20 μg/mL)^23^,^24^.

## Discussion

This study is the first to confirm that ART inhibited iris and retinal NV of rabbits by downegulating the expression of VEGFR2, PKCα, and PDGFR, and alleviated macular edema in monkeys with greater antrerior chamber penetrability and more durable efficacy than Avastin.

ART has been reported to inhibit corneal NV in animal models by inducing ROS-dependent apoptosis^22^. However, to date there have been no studies investigating the effect of ART on the iris or on retinal NV. We discovered that ART inhibited iris and retinal NV and alleviated macular edema in monkeys.

Furthermore, we proved that ART can attenuate retinal NV in rabbits by downregulating the expression of VEGFR2, PKCα, and PDGFR (Fig. 4, Fig. S2). VEGFR2 plays the most important role in promoting endothelial cell mitogenesis, angiogenesis, and permeability of retinal vessels^25^. Chen found that DI prevented angiogenesis by downregulating the levels of the VEGF receptors of human umbilical vein endothelial cells (HUVECs) in the concentration of 50 μM^20^. Similar effects were reported in lymphatic endothelial cells and Lewis lung carcinoma cells^19^. Our results were consistent with those of previous research. Downregulation of VEGFR2 might be a possible target of the anti-NV action of ART. PKCα is involved in the pathophysiological processes of DR^26^,^27^ and was also found to promote retinal pigment epithelial (RPE) cell proliferation in proliferative vitreoretinopathy (PVR) in our previous study^28^,^29^. The PKCα inhibitor midostaurin (PKC412A) has been used in a phase IIA study of metastatic melanoma^30^. PDGF was also demonstrated to be an important therapeutic target for the treatment of ocular NV^31^,^32^, and blocking PDGF pathways resulted in the inhibition of corneal NV^33^ and increased the effect of the VEGF pathway blockade by bevacizumab^34^. Therefore, ART blocked the multi-target of VEGFR2, PKCα, and PDGFR and therefore may partially address the problem of the limited efficacy of and resistance to anti-VEGF therapy alone.

In our research, the ocular NV of rabbits and monkeys was induced by intravitreal injection of VEGF_165_+bFGF, and vascular dilatation, tortuosity, and fluorescein leakage occurred in all groups at day 0 (Figs 2, 3 and 6). VEGF_165_ has the highest biological activity among the four main subtypes of VEGF^25^, and consequently the retinal NV induced by intravitreal injection of VEGF_165_+bFGF (which mimics the pathological progression of DR) in rabbits and monkeys has been used in many investigations^35^,^36^.

ART significantly inhibited retinal NV in rabbits and macular edema in monkeys with greater anterior chamber penetrability and more durable efficacy than Avastin and with on obviously toxic effect on eye structures and tissues during the follow-up period. ART had more durable efficacy (Figs 2 and 3), possibly because as a non-protein component, it has a more stable chemical structure than anti-VEGF protein drugs. Another reason might be that the multi-targeted inhibitory effect of ART can suppress NV more thoroughly than anti-VEGF drugs.

In addition, ART significantly inhibited iris NV and fluorescence leakage in the anterior chamber earlier and with greater anterior chamber penetrability (Figs 5 and 7). This might be because ART is a 384 Da molecule less than one-hundredth the size of Avastin, which is a 149 kDa full-length monoclonal antibody with an aqueous half-life of 9.82 days^37^.

In our monkey model, iris NV and macular edema were confirmed in a pathological study (Fig. 7A) and by OCT (Fig. 6E,F), but retinal NV and tractional retinal detachment did not occur. A higher dosage would induce severe and irreversible dense fluorescein leakage in the anterior chamber and would make observing the fundus difficult.

Evidence that shows VEGF is not the only target for anti-NV therapy is increasing. New molecular targets, such as bFGF, transforming growth factor (TGF)-β, angiopoietins, PKCα, and PDGF and their receptors, as well as other hypoxia-regulated gene products also provide avenues for improving the therapeutic benefit of anti-NV strategies^1^,^6^,^31^,^38^. Our results prove that ART can cover VEGF, PKCα, and PDGFR targets, with greater penetrability and more durable efficacy and may partially solve the issue of the limited efficacy of and resistance to anti-VEGF therapy alone.

In summary, our study is the first to confirm that ART inhibits iris and retinal NV. As an intravenous injection drug, ART might provide a totally different treatment strategy for AMD and DR after being formulated into eye drops and intravitreal injections, as has happened with Avastin.

## Methods

### Antibodies and Reagents

ART was obtained from Guilin Pharmaceutical Co., Ltd. (Guangxi, China). Avastin was obtained through Roche (Indianapolis, IN). VEGF R2 (ab10973, 1:400), PKCα (ab82540, 1:1000) and PDGFR (ab10848, 1:1000) were obtained from Abcam Biotechnology (Cambridge, Massachusetts, USA). β-actin (8H10D10) (#3700, 1:1000) Mouse antibody and secondary antibodies were purchased from Cell Signaling Technology (Boston, Massachusetts, USA).

### Animal Experiment

Sixty adult New Zealand white rabbits and 15 male cynomolgus monkeys were used in this study. The animals were anesthetized with intramuscular ketamine (30 mg/kg) and chlorpromazine hydrochloride (15 mg/kg) and euthanized by an overdose of lidocaine (25 mg/kg) administered by intravenous injection. The animals were housed under a 12 h: 12 h light–dark cycle with standard chow and water. Retinal NV in rabbit and monkey models was induced by VEGF_165_+bFGF treatment with a single intravitreal injection of ART and compared with Avastin.

### Retinal NV models and experimental protocols

Protocols of retinal NV models considered those from the published literature^35^,^36^ and were improved as described. Animals were placed under general anesthesia, and 0.5% proparacaine hydrochloride (Alcon-Couvreur, Puurs, Belgium) was applied topically for corneal anesthesia. Only the right eye of each animal was used. Pupils were dilated with 0.5% tropicamid (Shenyang Xingji Co., Shenyang, China) 15 min before intravitreal injection, which was performed after instillation of 1–2 drops of 5% povidine iodine in the conjunctival cul-de-sac. A lid speculum was used to keep the eyelids open, and the procedure was performed under direct visualization using a Zeiss operating microscope. The intravitreal injection was given according to the established method, taking all aseptic precautions.

Recombinant human VEGF_165_ (2 μg) and recombinant bFGF (2 μg) (Pepro Tech, Rocky Hill, NJ) diluted in 40 μL PBS with 0.1% BSA were injected into the vitreous of the right eyes using a 50-μL microsyringe (Hamilton, Switzerland) at the superior temporal quadrant, 3 mm behind the limbus, twice on 4 days and 2 days before the treatment, respectively. After the needle was withdrawn, the scleral entrance site was compressed with a cotton tip for 1 min; topical antibiotic ointment was instilled in the fornix.

Two days after the second injection of VEGF_165_+bFGF (day 0), the retinal vasculopathy was further evaluated with fundus photography and FFA. Grade +4 NV confirmed that models were successfully made (see below for grades of retinal NV).

The successful RNV model animals were randomly divided into three groups (model, ART, and Avastin) and were intravitreally treated with either normal sodium only or ART (20 μg), or Avastin (325 μg) for the corresponding group at day 0. All the left eyes served as normal controls. To ensure reproducibility, the same investigator performed the injections.

### Follow-up examinations

Follow-up examinations were performed on all monkeys and at least five rabbits from each group for 3 and 6 months, respectively, and included evaluation of the anterior segment by slit-lamp biomicroscopy (SL-D7; Topcon Co., Tokyo, Japan), corneal endothelial cell count (Topcon Co.), color fundus photography (TRC-50DX; Topcon Co.), FFA, optical coherence tomography (OCT, Stratus System, ver. 4.0 software; Carl Zeiss Meditec, Inc., Dublin, CA), and electroretinogram (ERG) (Roland, German). Enucleated rabbit eyes were processed for hematoxylin and eosin (H&E) staining and western blot at different time points (Table S1). Retinal thickness was measured at 2000 μm around the macula by OCT.

All fundus and FFA photographs of rabbits were evaluated independently by two observers in a masked manner and scored by using five grades of retinal NV^35^ as follows: Grade 0 displayed no vascular abnormalities in either the optic disk or the vascularized medullary rays. Grade +1 showed marked dilation and engorged tortuosity of the existing blood vessels in both the optic disk and medullary rays. Grade +2 displayed microvascular abnormalities, which presumably reflect new capillary buds. Grade +3 showed highly identifiable individual capillary loops growing into strands involving the optic disk and parts of the medullary rays. Grade +4 displayed total highly identifiable capillary loops growing into strands involving the entire optic disk and all of the medullary rays. Hemorrhaging occurred generally after Grade +4 was reached. Grade +5 showed the tractive fold of medullary fiber and tractional detachment of retina. The grade scores were used in statistical analysis.

### Quantification of retinal and anterior chamber fluorescein leakage

At each time point, at least five rabbits from each group and all the monkeys underwent FFA examination of both eyes. Eyes were dilated with topical 1% tropicamide, and the same fundus camera as used for color fundus photography was set for FFA with appropriate filters. We quickly injected 1 mL of 10% sodium fluorescein (10%, 0.1 mL; Alcon) for monkeys and 1 mL of 1% fluorescein (diluted with 1 mL of normal saline) for rabbits through a calf vein and marginal ear vein, respectively, to obtain fluorescein angiograms from 10 s to 15 min after injection. Sequential fundus photographs were taken immediately after fluorescein injection at 10 s, 30 s, and 1, 3, 5, and 15 min. At 15 minutes, anterior segment photographs were also taken. The retinal and aqueous chamber fluorescein leakage was quantified by measuring the fluorescein intensity in the selected areas using Image J (Figs 3B,C, 5C and 6C), and the mean fluorescein intensity was used in the statistical analysis.

### Oxygen saturation and diameter measurements of retinal vessels

The procedures for retinal vessel oxygen saturation and diameter measurements were performed as described in our previous study^39^ with the Oxymap T1 retinal oximeter (Oxymap T1; Oxymap ehf., Reykjavik, Iceland). A pseudocolor fundus image can provide a quick overview of the oxygen saturation of retinal vessels. Measurements were made from the inner circle (close to the edge of the optic disc) to the outer circle (500 pixels from the inner circle), as shown in Fig. S3L.

### Histopathology and immunohistochemical analyses

At each time point, three rabbits were sacrificed by injecting 7 mL lidocaine hydrochloride (PLATINUM, Guangzhou, China) through the marginal ear vein. The eyes were enucleated and fixed immediately in 4% paraformaldehyde solution for 48 h for histological and immunohistochemical analysis, or the retina were removed and immediately stored at −80 °C for protein extraction using western blot.

The retinal sections (5 μm) were heated in a sodium citrate buffer at 95 °C for 20 min for antigen retrieval. After naturally cooling to room temperature, the sections were blocked in 3% H_2_O_2_ for 15 min, then blocked in 5% BSA for 30 min at 37 °C, and then incubated with primary antibody at 37 °C for 2 h. After washing with PBS with Tween (PBST), the sections were incubated with HRP-conjugated secondary antibody at 37 °C for 20 min. The sections were then stained with diaminobenzidine (IHC kit, Abcam) for 30 s while being monitored under the microscope (Olympus BX53; Tokyo, Japan), followed by counterstaining with hematoxylin for 1 min and mounting with neutral balsam (No. 10160; Zhanyun Chemical Co. Ltd., Shanghai, China).

### Western blotting

At each time point, at least 3 rabbits from each group were sacrificed to extract retinal proteins from both eyes. The possible target proteins were analyzed by western blot analysis using specific primary antibodies. HRP-conjugated secondary antibodies were used to visualize the protein. The bands were detected using a diaminobenzidine detection kit (Boster Biotechnology, Wuhan, China). The same membrane was stripped and reprobed with an antibody specific to β-actin as the protein-loading control of total lysates. Stained bands were scanned, and intensity was quantified using the image analysis program Image J. Each test was repeated three times and the mean intensity was used in the analysis.

### Pilot Study

Following corneal anesthesia and mydriasis, intravitreal injection was performed after the instillation of 1–2 drops of 5% povidone iodine in the conjunctival cul-de-sac. We injected 80 μg ART from 3 mm behind the limbus at the superior temporal quadrant under direct visualization. After the needle was withdrawn, the scleral entrance site was compressed with a cotton tip for 1 min, and a topical antibiotic ointment was instilled in the fornix.

### Statistical Analysis

All data were reported as the mean ± standard deviation. Significant differences were determined using the *t* test or an ANOVA using SPSS 20.0 software (SPSS Inc. Chicago, IL, USA). Statistical analysis was also conducted using GraphPad Prism for Windows, version 5.00 (GraphPad Software Inc., La Jolla, CA, USA). Differences with a value of p < 0.05 were considered significant.

### Study approval

#### Animal study

Adult New Zealand white rabbits and cynomolgus monkeys were used for this study. All animals were treated in accordance with the Association for Research in Vision and Ophthalmology Statement for the Use of Animals in Ophthalmic and Vision Research. The protocol was approved by the Committee on the Ethics of Animal Experiments of Zhongshan Ophthalmic Center, Sun Yat-sen University (permit number: 2013-065 for rabbits and IACUU2013025 for monkeys).

#### Pilot Study

The study protocol was reviewed and approved by the Medical Ethics Committee of the Zhongshan Ophthalmic Center, Sun Yat-sen University (No. 2014MEK064), China, and adhered strictly to the principles of the World Medical Association Declaration of Helsinki. After receiving an explanation of the purpose and process of the study, the patient provided written informed consent before participating in the study.

## Additional Information

**How to cite this article**: Zong, Y. *et al*. Small Molecular-Sized Artesunate Attenuates Ocular Neovascularization via VEGFR2, PKCα, and PDGFR Targets. *Sci. Rep.* **6**, 30843; doi: 10.1038/srep30843 (2016).

## Supplementary Material

Supplementary Information

## References

[b1] Campochiaro P. A. Ocular neovascularization. J Mol Med 91, 311–321, doi: 10.1007/s00109-013-0993-5 (2013). 23329331 PMC3584193

[b2] Aiello L. P. . Vascular endothelial growth factor in ocular fluid of patients with diabetic retinopathy and other retinal disorders. N Engl J Med 331, 1480–1487, doi: 10.1056/NEJM199412013312203 (1994). 7526212

[b3] Ferrara N. & Kerbel R. S. Angiogenesis as a therapeutic target. Nature 438, 967–974, doi: Doi 10.1038/Nature04483 (2005). 16355214

[b4] Rosenfeld P. J. . Ranibizumab for neovascular age-related macular degeneration. N Engl J Med 355, 1419–1431, doi: 10.1056/NEJMoa054481 (2006). 17021318

[b5] Brown D. M. . Ranibizumab versus verteporfin for neovascular age-related macular degeneration. N Engl J Med 355, 1432–1444, doi: 10.1056/NEJMoa062655 (2006). 17021319

[b6] Carmeliet P. & Jain R. K. Molecular mechanisms and clinical applications of angiogenesis. Nature 473, 298–307, doi: 10.1038/nature10144 (2011). 21593862 PMC4049445

[b7] Tu Y. The discovery of artemisinin (qinghaosu) and gifts from Chinese medicine. Nat Med 17, 1217–1220, doi: 10.1038/nm.2471 (2011). 21989013

[b8] Qinghaosu Antimalaria Coordinating Research Group (Qinghaosu ACRG). Antimalaria studies on Qinghaosu. Chin Med J 92, 811–816 (1979). 117984

[b9] Crespo-Ortiz M. P. & Wei M. Q. Antitumor activity of artemisinin and its derivatives: from a well-known antimalarial agent to a potential anticancer drug. J Biomed Biotechnol 2012, 247597, doi: 10.1155/2012/247597 (2012). 22174561 PMC3228295

[b10] Haynes R. K. . C-10 ester and ether derivatives of dihydroartemisinin - 10-alpha artesunate, preparation of authentic 10-beta artesunate, and of other ester and ether derivatives bearing potential aromatic intercalating groups at C-10. Eur J Org Chem, 113–132, doi: 10.1002/1099-0690(20021) (2002).

[b11] Klayman D. L. Qinghaosu (artemisinin): an antimalarial drug from China. Science 228, 1049–1055 (1985). 3887571 10.1126/science.3887571

[b12] de Vries P. J. & Dien T. K. Clinical pharmacology and therapeutic potential of artemisinin and its derivatives in the treatment of malaria. Drugs 52, 818–836 (1996). 8957153 10.2165/00003495-199652060-00004

[b13] Lee J., Zhou H. J. & Wu X. H. Dihydroartemisinin downregulates vascular endothelial growth factor expression and induces apoptosis in chronic myeloid leukemia K562 cells. Cancer Chemother Pharmacol 57, 213–220, doi: 10.1007/s00280-005-0002-y (2006). 16075280

[b14] Zhou H. J., Wang W. Q., Wu G. D., Lee J. & Li A. Artesunate inhibits angiogenesis and downregulates vascular endothelial growth factor expression in chronic myeloid leukemia K562 cells. Vascul Pharmacol 47, 131–138, doi: 10.1016/j.vph.2007.05.002 (2007). 17581794

[b15] Chen H. H., Zhou H. J. & Fang X. Inhibition of human cancer cell line growth and human umbilical vein endothelial cell angiogenesis by artemisinin derivatives *in vitro*. Pharmacol Res 48, 231–236 (2003). 12860439 10.1016/s1043-6618(03)00107-5

[b16] Chen H. . Artesunate inhibiting angiogenesis induced by human myeloma RPMI8226 cells. Int J Hematol 92, 587–597, doi: 10.1007/s12185-010-0697-3 (2010). 20945119

[b17] Anfosso L., Efferth T., Albini A. & Pfeffer U. Microarray expression profiles of angiogenesis-related genes predict tumor cell response to artemisinins. Pharmacogenomics J 6, 269–278, doi: 10.1038/sj.tpj.6500371 (2006). 16432535

[b18] Zhu X. X. . Effects of sesquiterpene, flavonoid and coumarin types of compounds from Artemisia annua L. on production of mediators of angiogenesis. Pharmacol Rep 65, 410–420 (2013). 23744425 10.1016/s1734-1140(13)71016-8

[b19] Chen H. H., Zhou H. J., Wang W. Q. & Wu G. D. Antimalarial dihydroartemisinin also inhibits angiogenesis. Cancer Chemother Pharmacol 53, 423–432, doi: 10.1007/s00280-003-0751-4 (2004). 15132130

[b20] Chen H. H., Zhou H. J., Wu G. D. & Lou X. E. Inhibitory effects of artesunate on angiogenesis and on expressions of vascular endothelial growth factor and VEGF receptor KDR/flk-1. Pharmacology 71, 1–9, doi: 10.1159/000076256 (2004). 15051917

[b21] Huan-huan C., Li-Li Y. & Shang-Bin L. Artesunate reduces chicken chorioallantoic membrane neovascularisation and exhibits antiangiogenic and apoptotic activity on human microvascular dermal endothelial cell. Cancer Lett 211, 163–173, doi: 10.1016/j.canlet.2004.03.014 (2004). 15219940

[b22] Cheng R. . The artemisinin derivative artesunate inhibits corneal neovascularization by inducing ROS-dependent apoptosis in vascular endothelial cells. Invest Ophthalmol Vis Sci 54, 3400–3409, doi: 10.1167/iovs.12-11068 (2013). 23611999 PMC5963000

[b23] Lai H., Sasaki T. & Singh N. P. Targeted treatment of cancer with artemisinin and artemisinin-tagged iron-carrying compounds. Expert Opin Ther Tar 9, 995–1007, doi: Doi 10.1517/14728222.9.5.995 (2005). 16185154

[b24] Berger T. G. . Artesunate in the treatment of metastatic uveal melanoma–first experiences. Oncol Rep 14, 1599–1603 (2005). 16273263

[b25] Ferrara N., Gerber H. P. & LeCouter J. The biology of VEGF and its receptors. Nat Med 9, 669–676, doi: 10.1038/nm0603-669 (2003). 12778165

[b26] Geraldes P. & King G. L. Activation of protein kinase C isoforms and its impact on diabetic complications. Circ Res 106, 1319–1331, doi: 10.1161/CIRCRESAHA.110.217117 (2010). 20431074 PMC2877591

[b27] Fisher E. J., McLennan S. V., Yue D. K. & Turtle J. R. Cell-associated proteoglycans of retinal pericytes and endothelial cells: modulation by glucose and ascorbic acid. Microvasc Res 48, 179–189, doi: 10.1006/mvre.1994.1048 (1994). 7854204

[b28] Gao Q. Y. . PKC alpha affects cell cycle progression and proliferation in human RPE cells through the downregulation of p27(kip1). Mol Vis 15, 2683–2695 (2009). 20011080 PMC2791041

[b29] Gao Q. . The inhibitory effect of small interference RNA protein kinase C-alpha on the experimental proliferative vitreoretinopathy induced by dispase in mice. Int J Nanomedicine 8, 1563–1572, doi: 10.2147/IJN.S37635 (2013). 23626468 PMC3632628

[b30] Millward M. J. . The multikinase inhibitor midostaurin (PKC412A) lacks activity in metastatic melanoma: a phase IIA clinical and biologic study. Br J Cancer 95, 829–834, doi: 10.1038/sj.bjc.6603331 (2006). 16969355 PMC2360547

[b31] Nissen L. J. . Angiogenic factors FGF2 and PDGF-BB synergistically promote murine tumor neovascularization and metastasis. J Clin Invest 117, 2766–2777, doi: 10.1172/JCI32479 (2007). 17909625 PMC1994630

[b32] Dong A. . Antagonism of PDGF-BB suppresses subretinal neovascularization and enhances the effects of blocking VEGF-A. Angiogenesis 17, 553–562, doi: 10.1007/s10456-013-9402-5 (2014). 24154861 PMC3999311

[b33] Dell S., Peters S., Muther P., Kociok N. & Joussen A. M. The role of PDGF receptor inhibitors and PI3-kinase signaling in the pathogenesis of corneal neovascularization. Invest Ophthalmol Vis Sci 47, 1928–1937, doi: 10.1167/iovs.05-1071 (2006). 16639000

[b34] Perez-Santonja J. J., Campos-Mollo E., Lledo-Riquelme M., Javaloy J. & Alio J. L. Inhibition of corneal neovascularization by topical bevacizumab (Anti-VEGF) and Sunitinib (Anti-VEGF and Anti-PDGF) in an animal model. Am J Ophthalmol 150,519–528 e511, doi: 10.1016/j.ajo.2010.04.024 (2010). 20591397

[b35] Wong C. G., Rich K. A., Liaw L. H. L., Hsu H. T. & Berns M. W. Intravitreal VEGF and bFGF produce florid retinal neovascularization and hemorrhage in the rabbit. Curr Eye Res 22, 140–147, doi: DOI 10.1076/ceyr.22.2.140.5528 (2001). 11402391

[b36] Ozaki H. . Intravitreal sustained release of VEGF causes retinal neovascularization in rabbits and breakdown of the blood-retinal barrier in rabbits and primates. Exp Eye Res 64, 505–517, doi: 10.1006/exer.1996.0239 (1997). 9227268

[b37] Krohne T. U., Eter N., Holz F. G. & Meyer C. H. Intraocular pharmacokinetics of bevacizumab after a single intravitreal injection in humans. Am J Ophthalmol 146, 508–512, doi: 10.1016/j.ajo.2008.05.036 (2008). 18635152

[b38] Miller J. W. Vascular endothelial growth factor and ocular neovascularization. Am J Pathol 151, 13–23 (1997). 9212726 PMC1857918

[b39] Zong Y. . Retinal vessel oxygen saturation and vessel diameter in retinitis pigmentosa at various ages. Graefes Arch Clin Exp Ophthalmol, doi: 10.1007/s00417-015-3039-6 (2015). 25952041

