# Supplementary material for: Small Molecular-Sized Artesunate Attenuates Ocular Neovascularization via VEGFR2, PKCα, and PDGFR Targets
Source: Sci Rep. 2016 Aug 2;6:30843. doi: 10.1038/srep30843 (PMC4969591; doi:10.1038/srep30843)
Supplement: Supplementary Information [file srep30843-s1.pdf]

Title: Small Molecular-Sized Artesunate Attenuates Ocular Neovascularization via VEGFR2, PKCα, and PDGFR Targets

Authors:

Yao Zong, Yongguang Yuan, Xiaobing Qian , Zhen Huang, Wei Yang, Leilei Lin, Qishan Zheng , Yujie Li, Huining He,*Qianying Gao

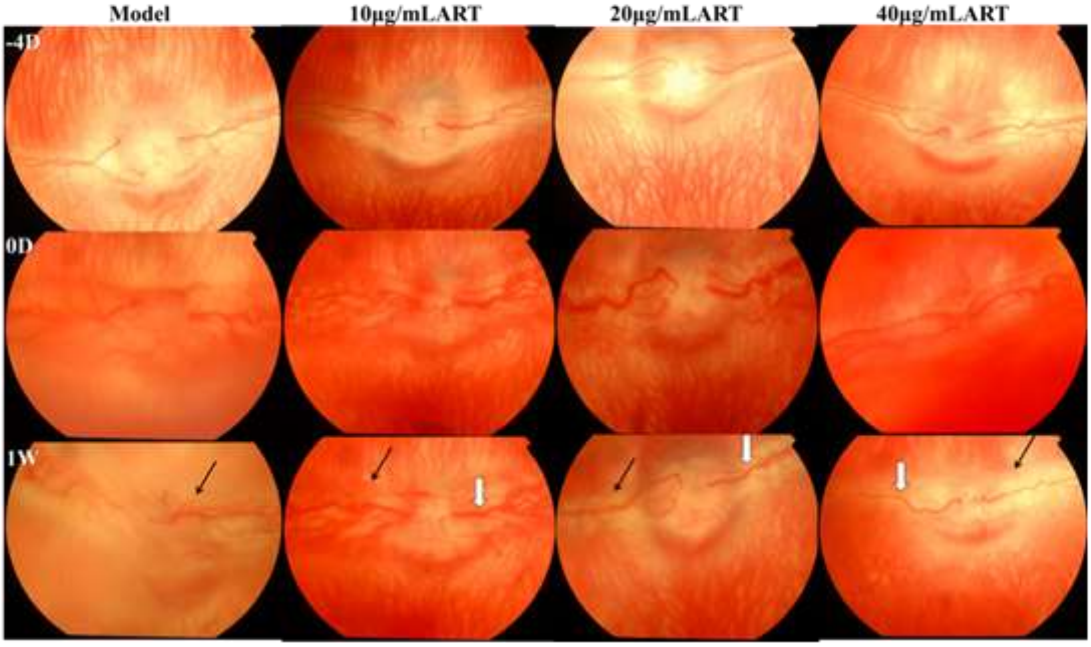

Figure S1. Screening effective dosage for the retinal NV model induced by VEGF165+bFGF

Intravitreal injection of 20 μg/mL and 40 μg/mL ART instead of 10 μg/mL ART can notably inhibit vascular dilatation and tortuosity (**white arrows**) and NV (**black arrows**) at the margin of medullary wings 1 week after treatment.

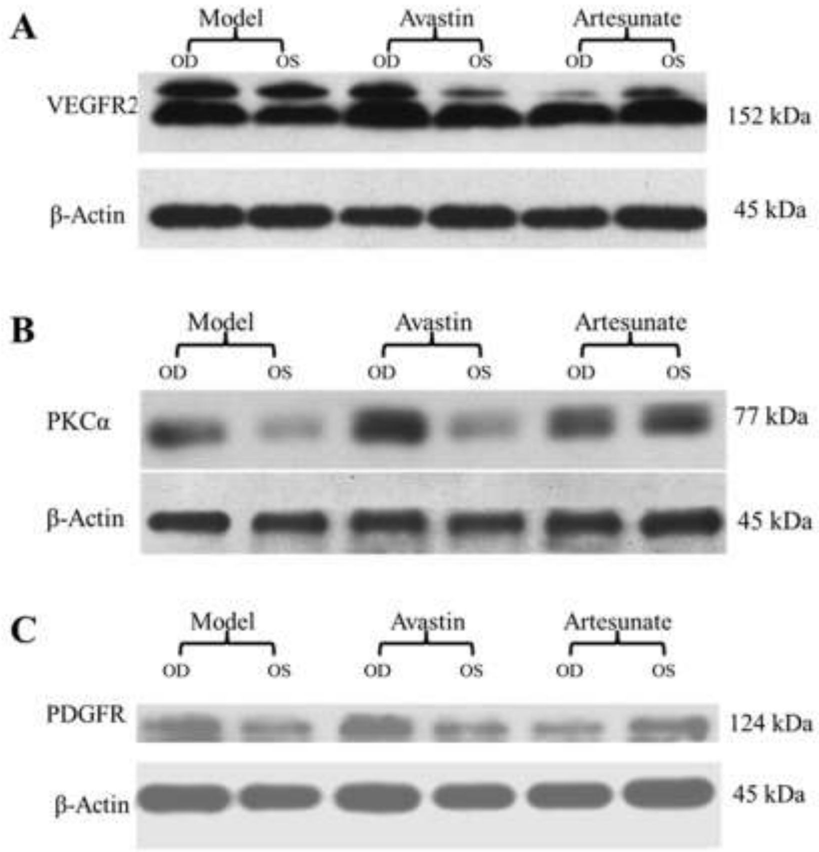

Figure S2. Western Blotting of rabbit retina tissue

Three rabbits from each group were harvested for Western blotting at 1 month. The expression of VEGFR2, PKCα, and PDGFR in the treated eyes of the Model and Avastin groups was stronger compared that in the contralateral eyes; however, the expression was significantly lower in the ART group.

The names of proteins are shown on the left, and the molecular weight sizes of proteins are shown on the right, respectively.

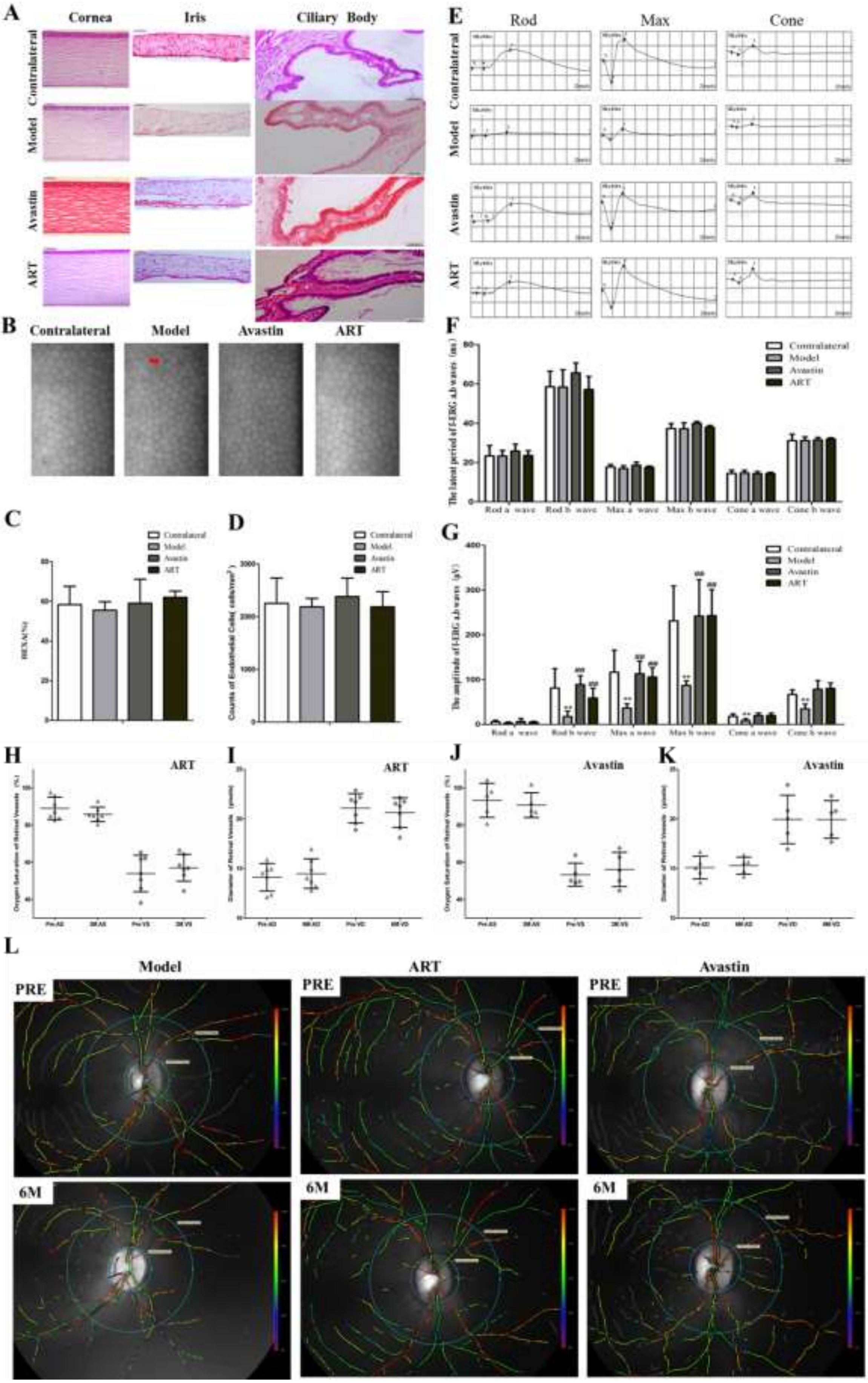
A
Cornea
Iris
Ciliary Body
Contralateral
Model
Avastin
ART
B
Contralateral
Model
Avastin
ART
C
D
E
Rod
Max
Cone
F
Contralateral
Model
Avastin
ART
Rod a wave
Rod b wave
Max a wave
Max b wave
Cone a wave
Cone b wave
G
H
ART
I
ART
J
Avastin
K
Avastin
L
Model
ART
Avastin
PRE
6M

Figure S3. **Safety evaluation of intravitreal injection of ART.**

A: The eye structure and tissue in rabbits treated with ART appeared normal without obvious pathologic change during the follow-up period.

B: No significant differences were found between the three groups and the contralateral eyes. Black corneal endothelial punctate defects (**red arrows**) were only found in the model group.

C-D: The cell density (CD） and percentage of hexagonal cells (HEXA) of monkeys were measured at the end of the follow-up period, and no significant differences were found between the three groups and the contralateral eyes.

E-G: The amplitude of the f-ERG a, b waves of the model group decreased significantly when compared with contralateral eyes and were much improved in the ART and Avastin groups. (* and ** denote $p<0.05$ and $p<0.01$ compared to the control group; # and ## denote $p<0.05$ and $p<0.01$ compared to the model group; $ and $$ denote $p<0.05$ and $p<0.01$ compared to the Avastin group.)

H-L: No significant differences were found in the diameter and oxygen saturation of retinal vessels in eyes treated by ART (**H-I**) or Avastin (**J-K**) after 6 months compared with levels before intravitreous VEGF165+bFGF.

Significant differences were determined using a t-test or an ANOVA with SPSS 20.0 software, differences with a value of $p<0.05$ were considered significant.

Table S1. Time points of follow-up examinations.

	Time points								
	-4D	0D	3D	1W	2W	4W	8W	12W	24W
Color fundus photography	+*	+*	+*	+*	+	+*	+*	+*	+
Slit-lamp biomicroscopy,	+*	+*	+*	+*	+	+*	+*	+*	+
FFA	+*	+*	+*	+*	+	+*	+*	+*	+
Corneal endothelial cell count									*
Histopathology				+		+			+
Western blot						+			+
OCT									*
ERG									*

+, rabbit; *, monkey
